# Processing emotion from abstract art in frontotemporal lobar degeneration

**DOI:** 10.1016/j.neuropsychologia.2015.12.031

**Published:** 2016-01-29

**Authors:** Miriam H. Cohen, Amelia M. Carton, Christopher J. Hardy, Hannah L. Golden, Camilla N. Clark, Phillip D. Fletcher, Kankamol Jaisin, Charles R. Marshall, Susie M.D. Henley, Jonathan D. Rohrer, Sebastian J. Crutch, Jason D. Warren

**Affiliations:** Dementia Research Centre, UCL Institute of Neurology, Department of Neurodegenerative Disease, University College London, London, United Kingdom

**Keywords:** Art, Emotion, Semantic dementia, Frontotemporal lobar degeneration

## Abstract

Abstract art may signal emotions independently of a biological or social carrier: it might therefore constitute a test case for defining brain mechanisms of generic emotion decoding and the impact of disease states on those mechanisms. This is potentially of particular relevance to diseases in the frontotemporal lobar degeneration (FTLD) spectrum. These diseases are often led by emotional impairment despite retained or enhanced artistic interest in at least some patients. However, the processing of emotion from art has not been studied systematically in FTLD. Here we addressed this issue using a novel emotional valence matching task on abstract paintings in patients representing major syndromes of FTLD (behavioural variant frontotemporal dementia, *n*=11; sematic variant primary progressive aphasia (svPPA), *n*=7; nonfluent variant primary progressive aphasia (nfvPPA), *n*=6) relative to healthy older individuals (*n*=39). Performance on art emotion valence matching was compared between groups taking account of perceptual matching performance and assessed in relation to facial emotion matching using customised control tasks. Neuroanatomical correlates of art emotion processing were assessed using voxel-based morphometry of patients' brain MR images. All patient groups had a deficit of art emotion processing relative to healthy controls; there were no significant interactions between syndromic group and emotion modality. Poorer art emotion valence matching performance was associated with reduced grey matter volume in right lateral occopitotemporal cortex in proximity to regions previously implicated in the processing of dynamic visual signals. Our findings suggest that abstract art may be a useful model system for investigating mechanisms of generic emotion decoding and aesthetic processing in neurodegenerative diseases.

## Highlights

• Abstract art may signal emotions independently of a biological carrier.
• Processing of emotion from art has not been studied systematically in FTLD.
• We studied this using a novel emotion valence matching task on abstract paintings.
• Versus controls all major FTLD syndrome groups showed an art emotion deficit.
• Art emotion decoding correlated with grey matter in lateral occipitotemporal cortex.

## 1 Introduction

Impaired processing of emotions has been widely documented in the frontotemporal lobar degenerations (FTLD). Emotional impairment is a defining feature of behavioural variant frontotemporal dementia (bvFTD: Rascovsky et al., 2011), generally prominent in the semantic variant of primary progressive aphasia (svPPA) and increasingly recognised in the nonfluent variant of primary progressive aphasia (nfvPPA; Kumfor and Piguet, 2012; Kumfor et al., 2013; Couto et al., 2013). Emotion deficits in FTLD are diverse and multidimensional: deficits may affect the cognitive processing of emotional cues in the verbal, visual, auditory or chemosensory modalities and extend across emotion categories (Snowden et al., 2001, Keane et al., 2002, Rosen et al., 2002, Werner et al., 2007, Bedoin et al., 2009, Omar et al., 2011a, Omar et al., 2011b, Kumfor et al., 2011, Rohrer et al., 2012, Hsieh et al., 2012a, Kumfor and Piguet, 2012) and the processing of both elementary emotions and more complex prosocial sentiments (Moll et al., 2011). Impaired processing of emotional signals is often mirrored by abnormal emotional behaviours exhibited by patients themselves and correlates both with impaired understanding of others' mental states (Snowden et al., 2001, Kipps et al., 2009, Shany-Ur et al., 2012) and with altered autonomic reactivity (Werner et al., 2007, Eckart et al., 2012, Balconi et al., 2015), consistent with the targeting of distributed neural networks that process emotion by the proteinopathies that underpin FTLD (Omar et al., 2011a, Omar et al., 2011b; Virani et al., 2013; Woolley et al., 2015). Limited evidence suggests that particular emotion categories or emotion modalities may be differentially affected in FTLD (Snowden et al., 2001, Kumfor et al., 2013, Lindquist et al., 2014, Oliver et al., 2015). However, it remains unclear to what extent the various dimensions and stages of emotion processing are separately affected in these diseases (Miller et al., 2012).

Most previous studies of emotion processing in FTLD have focussed on emotions linked to animate objects (in particular, human facial and vocal expressions), reflecting the preeminent biological and social value of such stimuli. However, emotions are not necessarily tied to such objects; aside from the preeminent symbolic code of language, humans also use various kinds of abstract nonverbal patterns to convey emotions. The relations of such abstract emotion codes to biologically grounded emotions and the impact of brain disease on those codes have not been defined. One familiar example of such an emotion code is music. Deficits of music emotion processing have been described in FTLD and have structural and neuroanatomical correlates overlapping the brain mechanisms identified for other emotion channels (Omar et al., 2011b, Hsieh et al., 2012b, Downey et al., 2013, Agustus et al., 2015). However, evidence from the study of the healthy brain as well as in patients with focal brain damage and developmental disorders suggests that music may have evolved to represent surrogate mental states, with privileged access to brain reward circuitry and mechanisms in common with those afforded to biological signals (Clark et al., 2015). In neurobiological terms, it is therefore unclear whether music truly qualifies as an ‘abstract’ emotion code or might rather have acquired the status of a ‘biological’ object.

Another candidate abstract emotional stimulus is abstract (nonrepresentational) art. The abstract works of artists such as Rothko, Kandinsky and Pollock attest to the propensity of such abstract patterns to evoke strong emotional responses in many viewers. Functional neuroimaging work in the healthy brain has identified substrates for processing both representational and abstract art (Kawabata and Zeki, 2004, Vartanian and Goel, 2004): abstract art engages distributed brain networks implicated in aesthetic experience, and these networks interact at the interface of perceptual (visual association cortical), emotional and evaluative (insula, orbitofrontal cortical, subcortical) and semantic (antero-lateral and medial temporal) processing (Di Dio and Gallese, 2009, Cupchik et al., 2009, Kirk et al., 2009, Vessel et al., 2012, Nadal, 2013, Chatterjee and Vartanian, 2014). Abstract art might therefore constitute a test case with which to assess whether emotion deficits in FTLD depend on biological carriers such as faces or voices or might dissociate from such carriers, reflecting a more generic impairment of emotion decoding. However, the brain mechanisms that process emotion in art are not well specified and the effects of FTLD on those mechanisms remain largely unknown (Aviv, 2014). Aside from its neurobiological interest, this question has clinical resonance. The relative stability of aesthetic preferences for artworks in patients with both FTLD and Alzheimer's disease (Halpern et al., 2008, Halpern and O'Connor, 2013, Graham et al., 2013, Silveri et al., 2015) together with intriguing observations of heightened or emerging artistic interest and competence in patients with FTLD (Chatterjee, 2006, Seeley et al., 2008, Miller and Miller, 2013) raise the possibility that art might be an island of relatively preserved emotional awareness in these diseases.

Here we addressed this issue by comparing the evaluation of emotion from nonrepresentational visual art and facial expressions using a novel within-modality emotion matching procedure in patients with canonical FTLD syndromes relative to healthy older individuals. We assessed structural neuroanatomical correlates of emotion processing using voxel-based morphometry (VBM) of patients' brain MR images. Extrapolating from the collective previous evidence of other emotion modalities (Kumfor and Piguet, 2012), we hypothesised that patients with FTLD would have deficits of art emotion decoding and that these deficits would be relatively more prominent in patients with bvFTD and svPPA than with nfvPPA; but further, that these emotion deficits would dissociate between the modalities of art and facial expressions. In addition, we hypothesised that art and facial emotion impairments in FTLD would have separable neuroanatomical correlates within the large-scale brain network previously implicated in the processing of emotion from visual stimuli (Kawabata and Zeki, 2004, Vartanian and Goel, 2004, Di Dio and Gallese, 2009, Cupchik et al., 2009, Vessel et al., 2012).

## 2 Methods

### 2.1 Participants

Twenty-four patients fulfilling current consensus criteria for syndromes of frontotemporal lobar degeneration (11 with bvFTD: Rascovsky et al., 2011; seven with svPPA, six with nfvPPA: Gorno-Tempini et al., 2011) were recruited via a tertiary-level specialist cognitive disorders clinic. The clinical diagnosis was supported in each case by volumetric brain MRI showing a consistent profile of regional atrophy with no significant associated burden of cerebrovascular disease. Thirty-nine healthy older individuals with no history of neurological or psychiatric illness also participated in the study. No participant had a history of clinically significant peripheral visual impairment and none had been involved professionally in the visual arts. Characteristics of participant groups are summarised in Table 1.

### 2.2 Assessment of artistic background

A questionnaire assessing participants' previous art exposure and expertise was administered to patients' caregivers and to healthy control participants (Table S2 in Supplementary Material on-line). The questionnaire included measures of artistic training, current production of art and current level of interest in art. Items were scored on a Likert scale ranging from 0 (no practical experience or interest in viewing art) to 5 (high interest in viewing art and/or regular art production).

### 2.3 General neuropsychological assessment

All patients and a subset of 25 healthy controls had a comprehensive assessment of background neuropsychological functions (Table 1) including general intellect (Wechsler Abbreviated Scale of Intelligence (WASI), Wechsler, 1999; National Adult Reading Test, Nelson, 1982), executive skills (Stroop colour-reading, word-reading and interference conditions from the Delis-Kaplan Executive Functioning System (D-KEFS), Delis et al., 2001; Letter Fluency and Category Fluency subtests from the D-KEFS Verbal Fluency Test; D-KEFS Trail Making Test; Digit Symbol subtest from the Wechsler Adult Intelligent Scale Revised, Wechsler, 1987), working memory (digit span from the Wechsler Memory Scale Revised, Wechsler, 1987), visual apperception (Object Decision Subtest of the Visual Object and Space Perception (VOSP) Battery, Warrington and James, 1991), episodic memory (Recognition Memory Test, Warrington, 1984; Paired Associate Learning Test from the Camden Memory Tests, Warrington, 1996), semantic memory (British Picture Vocabulary Scale, Dunn et al., 1997), word retrieval (Graded Naming Test, McKenna and Warrington, 1983) and calculation (Graded Difficulty Arithmetic, Jackson and Warrington, 1986).

In order to provide a reference for interpreting performance profiles on the experimental tests, we assessed a subset of patients from each syndromic group (five bvFTD, three svPPA, three nfvPPA) on standard, widely available tests of colour perception and emotion identification. The test of colour perception (described by Shakespeare et al. (2013)) comprised items selected from the original Munsell colour system, participants were presented with 48 pairs of matt colour chips with fixed value and chroma, arranged into three levels of difficulty whereby with each advancing level, coloured pairs were increasingly similar in hue; the task on each trial was to discriminate whether the chips in each pair were of the same or different hue. The test of emotion identification (Omar et al., 2011a) comprised facial expressions of happiness, sadness, anger, and fear (10 for each emotion, 40 trials in total) derived from the canonical facial emotion pictures set of Ekman and Friesen (1976); stimuli were presented in random order and the task on each trial was to match each target stimulus with the most appropriate verbal emotion label in a four-alternative-forced-choice paradigm.

### 2.4 Experimental stimuli and procedures

#### 2.4.1 Assessment of emotion in art

**Fig. 1:**
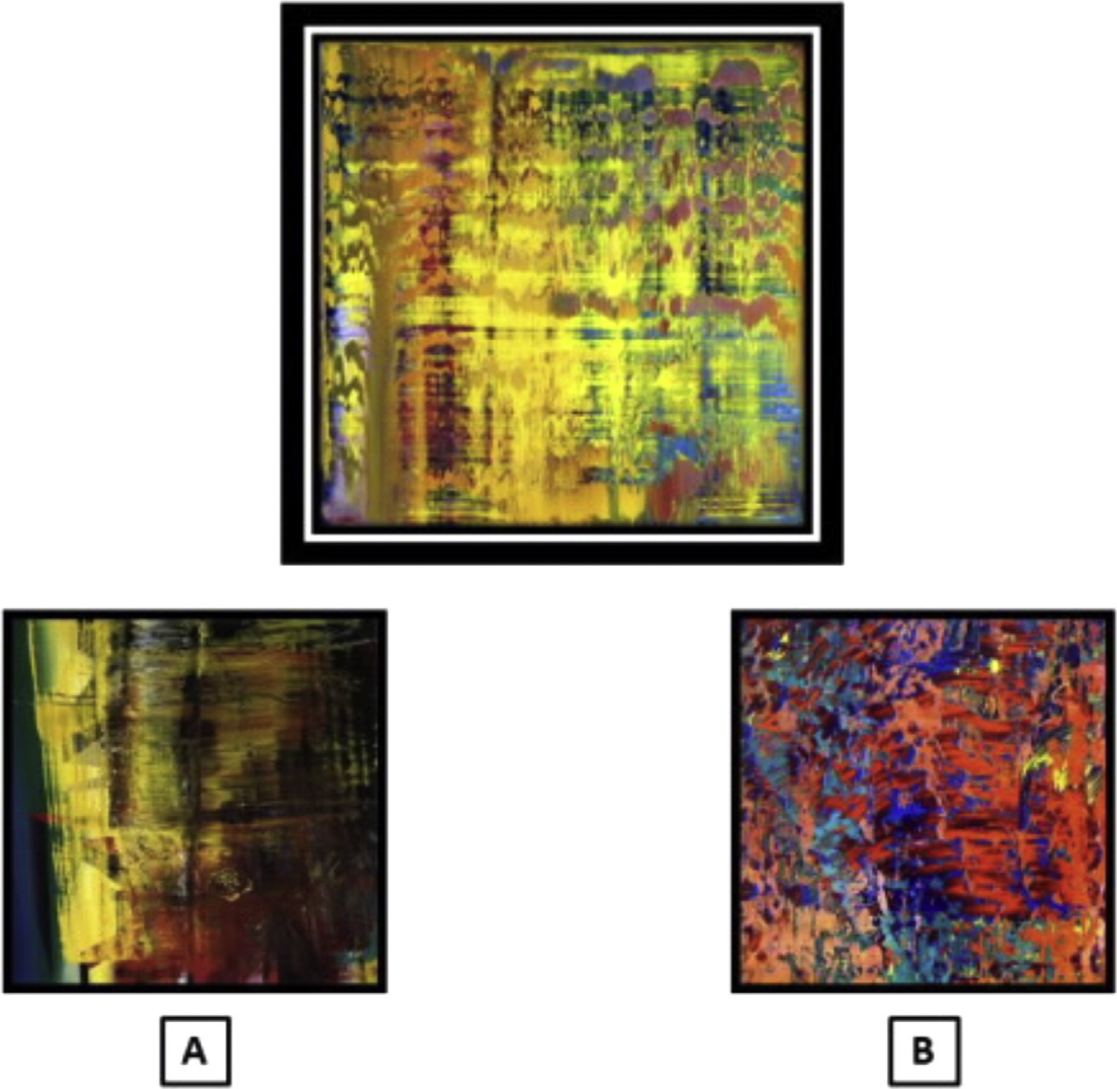
An example of a stimulus triad from the art emotion valence matching test (individual paintings have been adapted from the Richter originals for illustrative purposes). The probe stimulus is shown above; the foil and target stimuli are below. The probe stimulus here has ‘positive’ emotional valence based on pilot control ratings; the target (matching) stimulus here is B.

We created a test requiring classification and matching of the emotional valence of abstract paintings: the ‘art emotion’ test. Our chief objectives in this new test were to isolate emotion recognition processes that are largely free of previous associations with familiar objects in the world at large and do not depend on cross-modal (e.g., verbal) labelling. In order to generate the art stimulus set we first selected 60 digital images representing abstract works by the same artist (Gerhard Richter, born 1932) publically available on the World Wide Web. Richter's non-representational paintings are intensely abstract and renounce any overt links to objects in the world at large (see adapted examples in Fig. 1); they make extensive use of colour and organic lines and shapes that often suggest alien scenes or landscapes where any emotional tone grows incidentally from the surface characteristics of the paint layers. As such, Richter's works are ideally suited to evoke emotions that lack any prior semantic associations. In addition, his prolific oeuvre reduces the likelihood that particular works will be familiar to a non-specialist audience or will have acquired personal semantic associations.

In an initial pilot experiment, nineteen healthy younger adults (age range 23–57 years; ten female, nine male) with variable prior art and experience were presented with the 60 images and asked to provide an overall broad valence judgement (‘positive’ or ‘negative’) and a descriptive adjective for each painting. Overall valence judgments and adjectives were subsequently analysed and original spontaneous descriptions were recoded according to whether they were directly compatible with the positive or the negative valence category (e.g., ‘joyous’, ‘nervous’) or ambiguous (‘e.g., ‘autumnal’, ‘intense’). Thirty-two paintings achieving a consensus of descriptions (coded as either unambiguously positive or negative) by >75% of the pilot group comprised the final stimulus set used in the art emotion test.

The 32 images of this consensus set were rearranged to form 20 triads each comprising a probe image, a target image of matching emotional valence and a foil image of opposite emotional valence. On 10 trials, the probe image had positive emotional valence based on pilot control ratings; on the remaining 10 trials the probe had negative valence. Triads were composed such that matching of probe to target could not be achieved simply by matching surface similarities (such as dominant colour or structural features) across the stimulus set. Stimulus triads are listed in Table S1 and further stimulus details are provided in Supplementary Material on-line.

#### 2.4.2 Assessment of facial emotion

In order to compare emotion judgements from abstract art with emotion judgements via an animate visual emotion channel, we created a parallel test for the matching of facial expressions. We used stimuli from the ‘NimStim’ set (Tottenham et al., 2009; available on request from the authors at http://www.macbrain.org/resources.htm) in which the universal human facial expressions (happiness, sadness, anger, fear, disgust, surprise) are conveyed by a wide range of actors and actresses from different ethnic backgrounds. Twenty-four stimulus trials (four probing each of the six canonical expressions) were created; each trial comprised a triad of probe, target (matching) and foil facial expressions (see Fig. S1). Triads were composed such that matching of probe to target could not be achieved simply by matching surface similarities (such as gender or ethnic origin) across the stimulus set.

#### 2.4.3 Perceptual control test

**Fig. 2:**
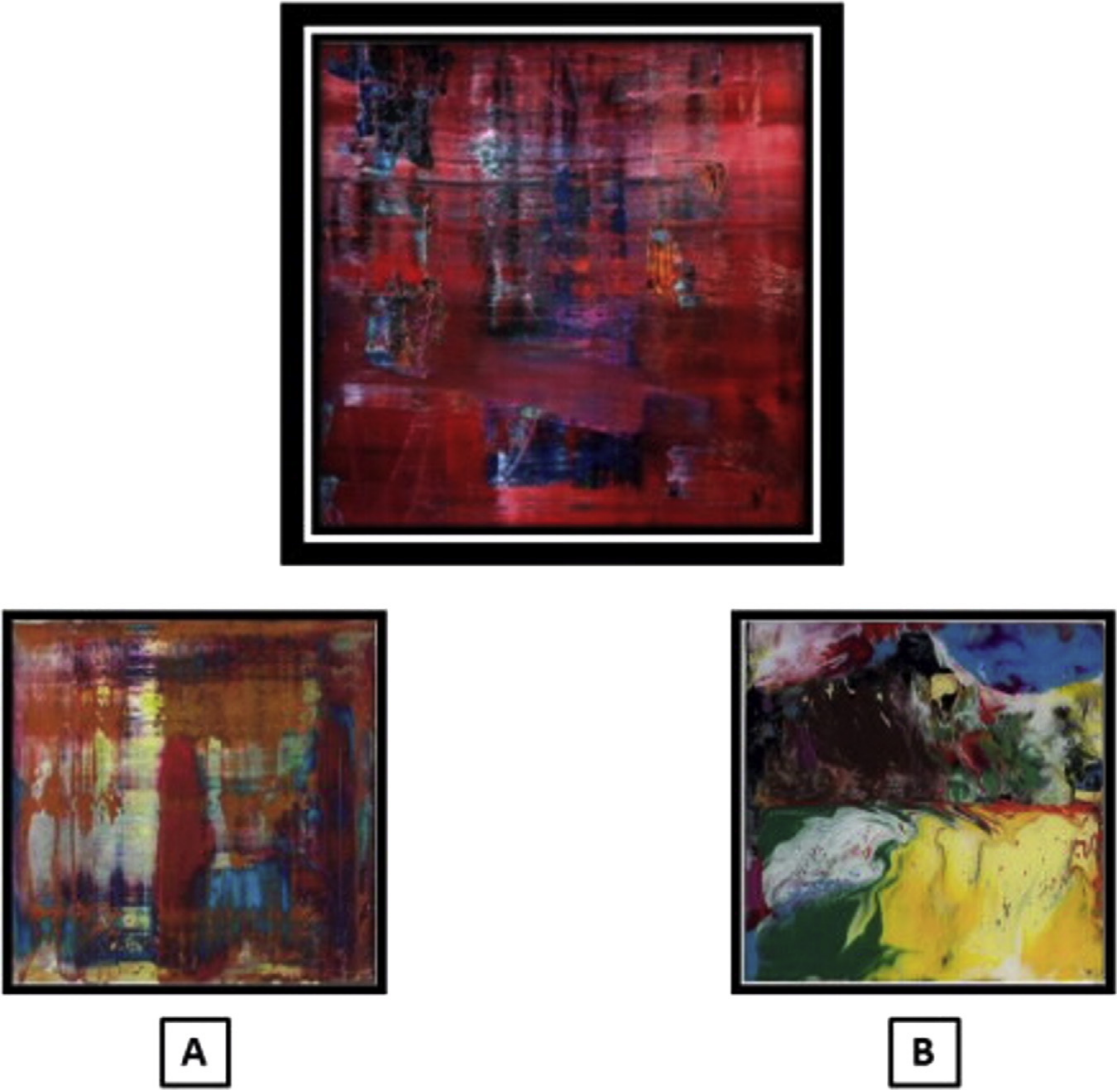
An example of a stimulus triad from the art perceptual matching control test (individual paintings have been adapted from the Richter originals for illustrative purposes). The probe stimulus is shown above; the foil and target stimuli are below. In this example, the target (matched to the probe for dominant hue) is A.

In addition to a judgment on emotional valence *per se*, the art emotion test here entailed perceptual analysis of complex visual stimuli and comparison of stimuli presented in an array. Accordingly, in order to control for these task demands and allow interpretation of any deficit of emotion processing, we created a customised perceptual matching control test on the same abstract art stimuli. Stimuli from the art emotion test were rearranged to comprise 20 triads in which the probe and target images were matched for dominant hue rather than emotional valence (see Fig. 2).

#### 2.4.4 Experimental protocol

Visual stimuli were presented as high resolution colour images on the monitor screen of a notebook computer (see Fig. 1). In the art emotion test, on each trial a probe image, a target image and a foil image were presented and the participant was asked to decide whether the target or the foil image more closely matched the probe stimulus in emotional valence, where valence was defined for participants as ‘positive/happy’ or ‘negative/unhappy’. The forced choice decision in the art emotion task was therefore based on two emotion categories. In the facial emotion test, an analogous procedure was used but the task was to decide whether the target or foil stimulus more closely matched the probe stimulus in emotional expression. The forced choice decision in the facial emotion task was therefore based on six emotion categories (the canonical facial expressions). In the art perceptual control test, on each trial the task was to decide whether the target or foil stimulus most closely matched the probe stimulus in overall colour. On each trial, the probe stimulus was presented above the target and foil images; the relative screen positions of target and foil stimuli were randomised between trials in each test.

Tests were administered in the order: art emotion, art perceptual control, facial emotion. Before each test participants were presented with practice examples to ensure they understood the task. For the emotion tests, it was emphasised that the task required a decision about the kind of emotion conveyed by each stimulus and to match stimuli for similar emotions, rather than on the basis of any particular feature or personal aesthetic preference. Trials were presented in randomised order in each test. Participant responses were recorded for offline analysis. During each test, no feedback about performance was given and no time limits were imposed.

### 2.5 Analysis of behavioural data

Behavioural data were analysed using SPSS^®^v22 and Stata12^®^. Demographic, general neuropsychological and art perceptual control test data were compared between participant groups using post-hoc analysis of variance (Games et al., 1979); as normality assumptions were violated, differences in categorical variables were assessed using Fisher's exact test and the small patient subsets completing the hue discrimination and adapted Ekman emotion identification tests were compared to healthy control norms using modified *t*-tests (Crawford and Garthwaite, 2002). Performance on the experimental emotion tests was initially compared between groups in a linear regression model with robust, clustered standard error: this model incorporated scores (as proportions of trials correct) on both the art and face emotion tests with age and gender as covariates of no interest, allowing us to compare performance between groups and between emotion modalities in a common statistical framework. In a subsequent analysis, we assessed the impact of potential perceptual and cognitive executive confounds on art emotion valence matching using a separate linear regression model that compared art emotion scores between groups incorporating age, gender, art perceptual control score and WASI Similarities score (an index of abstract reasoning) as covariates of no interest. In addition, correlation analyses based on Spearman's rho were performed to assess any relationship of performance on the art emotion test with the facial emotion and perceptual control tests as well as with pertinent background characteristics including previous art experience, symptom duration, WASI Similarities and VOSP scores. For all tests, a threshold *p*<0.05 was accepted as the criterion for statistical significance.

### 2.6 Voxel based morphometry

#### 2.6.1 MRI acquisition

Volumetric MR brain images were acquired for 18 patients (seven with bvFTD, six with svPPA, five with nfvPPA) on a Siemen's Trio 3T MRI scanner using a 32-channel phased array head-coil. T_1_-weighted images were obtained using a sagittal 3D magnetisation rapid gradient echo sequence (echo time/repetition time/inversion time=2.9/2200/900 ms, dimensions of 256×256×208, voxel size of 1.1×1.1×1.1 mm^3^).

#### 2.6.2 Image pre-processing

Pre-processing of patients' brain MR images was performed using the DARTEL toolbox within SPM12b (www.fil.ion.ucl.ac.uk/spm) running under MATLAB R2012a (www.matlab.com) Using the “Segment” routine in SPM12b, native-space whole-brain MR images were segmented into native-space grey matter, native-space white matter and cerebrospinal fluid and rigidly-aligned grey and white matter segments. Bias-corrected whole brains in native space were also output. The rigidly-aligned grey and white segments were used to create DARTEL templates with the “run DARTEL (create Templates)” command under DARTEL tools. Finally the “Normalise to MNI space” command was used with 1 mm isotropic voxel size to warp into MNI space, modulate and smooth (6 mm full width half-maximum) the grey matter segments prior to statistical analysis. The bias-corrected whole brain images in native space were also warped into MNI space (with 1 mm isotropic voxel size) using “Normalise to MNI space” and then averaged in order to create a study-specific template image on which to overlay the results for visual presentation. To help protect against voxel drop-out because of potentially marked local regional atrophy in particular scans, a customised explicit brain mask was derived by maximising the correlation between the binary mask and the average of images to be analysed (Ridgway et al., 2009) and applied to the images prior to analysis. In order to adjust for individual differences in head size and total grey matter, total intracranial volume was calculated for each participant by summing grey matter, white matter and cerebrospinal fluid volumes following segmentation of all three tissue classes.

#### 2.6.3 Image analysis

Using three separate linear regression models, voxel intensity (an index of grey matter volume) was assessed over the combined patient cohort as a function of score on the art emotion valence matching test, the art perceptual matching test or the face emotion matching test. Patient age, gender and total intracranial volume were included as covariates of no interest in all models. For each model, contrasts were applied to assess regional grey matter volume that was either positively associated (‘forward’ contrast) or negatively associated (‘reverse’ contrast) with performance on the test of interest over the patient cohort. Anatomical small volumes of interest based on the prior anatomical hypotheses were created to cover key regions in each cerebral hemisphere previously implicated in emotional analysis of art stimuli: these regions included visual areas in occipital and occipito-temporal cortices, insula and orbitofrontal cortex (Kawabata and Zeki, 2004, Vartanian and Goel, 2004, Di Dio and Gallese, 2009, Cupchik et al., 2009, Vessel et al., 2012). Regions were edited from the Harvard–Oxford histological brain maps in FSLview v3.1 to fit the mean brain template. Visual cortical regions comprised postero-ventral areas including V1, cuneus, lingual and posterior fusiform gyri and a more dorso-lateral occipito-temporal area including area V5 (all anatomical regions assessed are shown in Fig. S2 in Supplementary Material on-line). Statistical parametric maps of regional grey matter correlates of performance on the emotion and perceptual tests were examined at threshold *p*<0.05 after family-wise error (FWE) correction for multiple voxel-wise *t*-tests over the pre-specified anatomical regions of interest, assessed separately in each cerebral hemisphere. In addition, in order to assess regional variation of effects unthresholded over the whole brain, maps of effect size (parameter estimates) were created using the ‘slover’ command in SPM12b, overlaying beta images onto the customised template brain image.

## 3 Results

### 3.1 General characteristics of participant groups

Results of participant group comparisons are summarised in Table 1. Participant groups did not differ significantly in gender distribution (*p*=0.16), age (*p*=0.74) or background art experience (*p*=0.93); none of the healthy control participants recognised the artist or the artworks presented and patient caregivers similarly indicated that these would not have been familiar to any of the patients premorbidly. Patients with nfvPPA had significantly fewer years of education than other patient groups (*p*<0.05), however all participant groups were relatively highly educated. Symptom duration was significantly shorter in the nfvPPA group than other patient groups (*p*<0.05).

General neuropsychological findings corroborated the syndromic diagnoses for the patient groups (Table 1). Relative to healthy controls (*p*<0.05), all patient groups showed deficits of verbal fluency, other aspects of executive function and naming; while the bvFTD group showed deficits of both verbal and nonverbal episodic memory, the svPPA group had deficits of word memory and reading and the nfvPPA group had a marked deficit of polysyllabic word repetition. The svPPA group as anticipated had a particularly severe deficit of naming in relation to both other patient groups (*p*<0.05). No patient group showed a deficit of visual apperceptive function (as assessed using VOSP performance).

### 3.2 Performance on background perceptual and emotion processing tests

On the customised art perceptual control test (see Table 2), after adjusting for age and gender the nfvPPA group performed significantly worse than the healthy control group (beta=−0.34, *p*<0.05); performance of the other patient groups did not differ significantly from healthy controls (bvFTD, beta=−0.26, *p*=0.09; svPPA, beta=−0.16, *p*=0.31) and there were no significant differences between patient groups (*p*>0.05). On the hue discrimination test (see Table 1), the patient cohort showed no significant overall deficit relative to healthy control norms (*t*_(10)_=−1.7, *p*=0.12; Shakespeare et al., 2013); however, one patient with bvFTD and two patients with nfvPPA showed a deficit on this test (score>2 standard deviations below healthy control mean). On the adapted Ekman facial emotion identification test, the patient cohort showed a significant overall deficit relative to healthy control norms (*t*_(10)_=−3.9, *p*<0.01; Omar et al., 2011a); all patients with bvFTD, one patient with svPPA and two patients with nfvPPA showed a deficit on this test (score>2 standard deviations below healthy control mean).

### 3.3 Performance on emotion matching tests

Group performance profiles for all experimental tests are summarised in Table 2; plots of individual data on the art emotion valence matching test are presented in Fig. S3 in Supplementary Material on-line. Two healthy control participants performed as outliers on the art emotion task (based on individual *z* scores >2 standard deviations from the control group mean) and data from these participants were excluded from the main analysis (a posthoc reanalysis including these individuals left the results unaltered).

The initial comparison of groups and emotion tasks, revealed a significant main effect of group (*F*_(3,60)_=20.28, *p*<0.01) but no significant main effect of emotion modality (*F*_(1,60)_=2.44, *p*=0.12) and no significant interaction between group and emotion modality (*F*_(3,60)_=1.67, *p*=0.18). Across emotion modalities, all patient groups performed significantly worse than the healthy control group (bvFTD, beta=−0.23, *p*<0.01; svPPA, beta=−0.13, *p*<0.01; nfvPPA, beta=−0.17, *p*<0.01); the bvFTD group showed borderline significantly worse performance than the svPPA group (beta=−0.10, *p*=0.05), but there were no other significant differences between patient groups.

In the separate comparison of art emotion valence matching between groups taking background cognitive (art perceptual control and abstract reasoning) factors into account, there remained a significant main effect of group (*F*_(7, 32)_=4.15, *p*<0.01), each patient group showing significantly worse art emotion valence performance than healthy controls (bvFTD, beta=−0.73, *p*<0.01; svPPA, beta=−0.61, *p*<0.01; nfvPPA, beta=−0.41, *p*<0.05); neither art perceptual control or WASI Similarities performance significantly predicted performance on the art emotion valence matching task (*p*>0.05).

Performance on art emotion valence matching was not significantly correlated with facial expression matching, performance on the art perceptual control task, background art experience, symptom duration nor standard measures of abstract reasoning or visuoperceptual performance (WASI Similarities score, VOSP score; all *p*>0.05).

### 3.4 Neuroanatomical associations

**Fig. 3:**
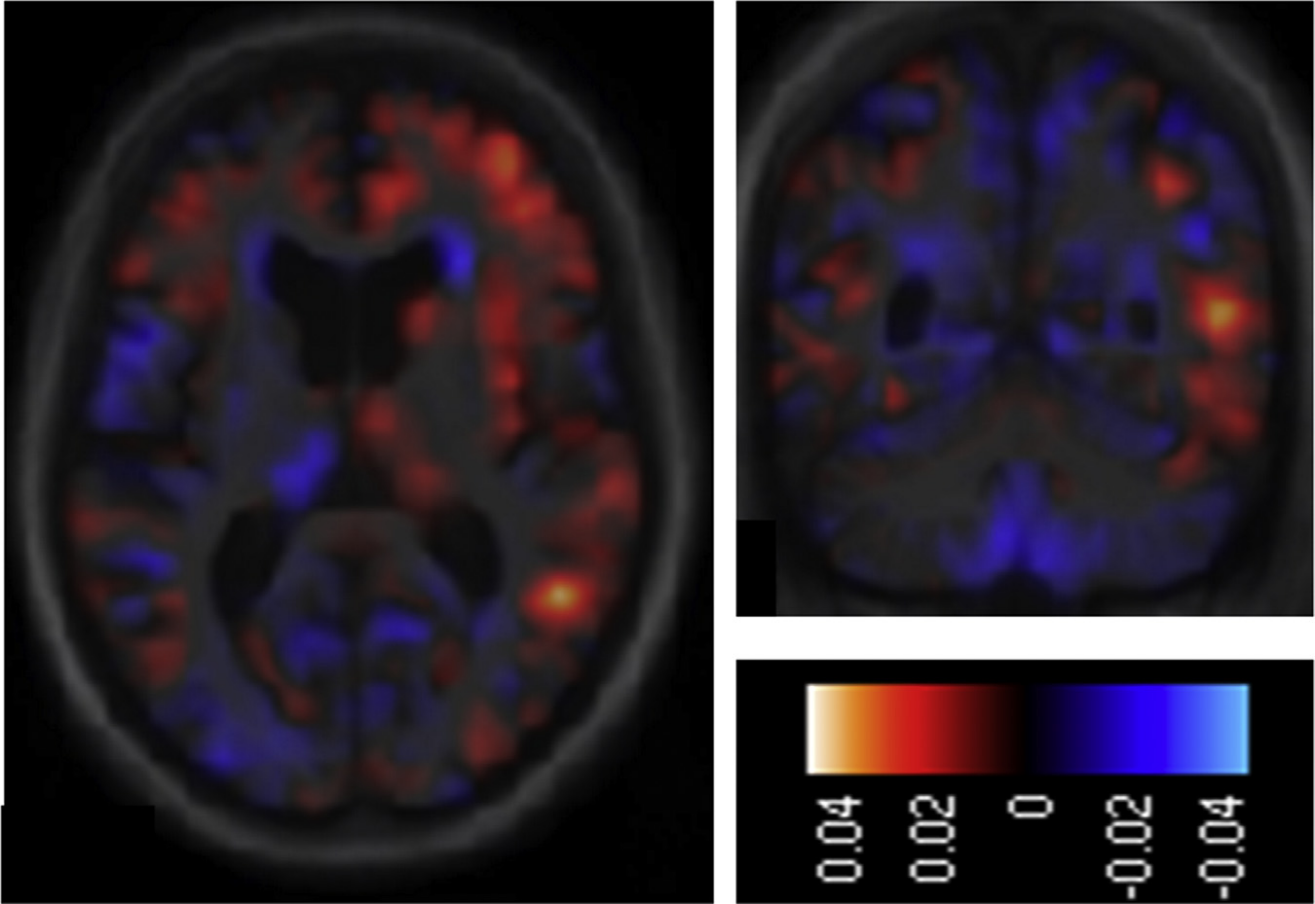
Maps of effect (parameter estimate) size for the art emotion valence matching contrast in the combined patient cohort. The colour bar codes the value of the parameter estimate. Maps are shown on representative axial (left) and coronal (right) sections of the group mean template brain image, selected to demonstrate the peak correlate in right temporo-occipital association cortex; the right hemisphere is shown on the right. These effects size maps are unthresholded however the peak correlate shown was significant at threshold *p*<0.05_FWE_ after correction for multiple voxel-wise *t*-tests within the prespecified anatomical region of interest (see text).

The VBM analysis of the combined patient cohort revealed a significant positive association between art emotion valence matching score and grey matter volume in right lateral occipito-temporal cortex (MNI peak coordinates [39 −72 −3]; *p*<0.05_FWE_ after correction for multiple voxel-wise comparisons within the pre-specified anatomical volume of interest). No other significant grey matter associations of art emotion processing were identified at the prescribed threshold in any of the regional volumes examined. However, maps of effect size demonstrated sub-threshold regional variation of grey matter volume positively associated with performance on the art emotion task within a network of areas extending anteriorly from temporo-occipital cortex into antero-mesial temporal lobe, temporal pole and orbitofrontal cortex, preponderantly in the right cerebral hemisphere (Fig. 3). No significant grey matter associations of art perceptual processing or facial emotion processing were identified at the prescribed threshold.

## 4 Discussion

Here we have shown that (relative to healthy older individuals) patients with canonical syndromes of FTLD have impaired processing of emotion conveyed by nonrepresentational visual art. This deficit of art emotion processing was shown by each of the FTLD syndromic groups assessed. Consistent with previous evidence (Kumfor and Piguet, 2012, Rohrer et al., 2012), patients in each group showed deficits on a standard measure of facial emotion identification. Perhaps more surprisingly, patients with nfvPPA here also showed deficits on a standard measure of colour perception and impaired perceptual matching of visual art. However, all patient groups showed impaired processing of emotion from art after adjusting for performance on the control visual perceptual matching task, which was closely matched for stimulus and response characteristics: it is therefore unlikely that impaired art emotion valence matching in the patient cohort was driven primarily by perceptual factors. Moreover, within the patient cohort impaired art emotion processing was not correlated with face emotion processing, perceptual task performance, general disease stage or severity factors nor with previous artistic background. Taken together the present evidence suggests that major FTLD syndromes produce dysfunction of neural emotion mechanisms that are accessed by abstract art. This work adds to the substantial body of previous evidence for deranged emotion processing in FTLD (Snowden et al., 2001, Keane et al., 2002, Rosen et al., 2002, Werner et al., 2007, Bedoin et al., 2009, Omar et al., 2011a, Omar et al., 2011b, Kumfor et al., 2011, Rohrer et al., 2012, Hsieh et al., 2012a, Hsieh et al., 2012b, Kumfor and Piguet, 2012). Our findings build on this previous corpus by demonstrating that impairments of emotion processing in these diseases do not depend strongly on biological carriers or social signalling *per se*: rather, the emotional deficit in FTLD extends to the decoding of emotion from abstract patterns. Indeed, the abstract patterns of visual art lack even the social associations of music: while it is also undeniably a symbolic code of high emotional value, music is typically embedded in a social context and well known to engage the social brain (Clark et al., 2015). Abstract art may engage brain mechanisms that support the generic decoding of emotional signals (Aviv, 2014).

In line with this formulation, we found no evidence of an interaction between group performance and the modality of emotion matching. On the other hand, the lack of correlation between emotion modalities argues against any strong claim to equivalence between emotion modalities. These findings should be interpreted cautiously, due both to the relatively small case numbers here (which will have limited power to detect weaker effects) and the emotion matching paradigm itself. This paradigm was designed to equate task procedures for facial expressions and paintings while minimising any requirement for verbal or cross-modal labelling but has not been widely used in previous studies of FTLD. Moreover, the task requirements of emotion matching may not have been neuropsychologically equivalent for art and facial expressions. Whereas the art emotion matching test here required an assessment of overall emotional valence between stimuli, the facial emotion matching test required a more specific mapping to particular emotional expressions. Furthermore, facial expressions are constituted by universal feature configurations learned during normal development whereas it is most unlikely that specific semantic associations were previously learned for the artworks presented here. It is therefore possible that the facial emotion matching task engaged apperceptive mechanisms that were not available for the art emotion valence matching task. The most parsimonious interpretation may be that mechanisms of emotion processing engaged by art and facial expression are partly shared but that certain aspects of the processing of emotion via each channel may engage mechanisms not engaged by the other emotion channel. If this is the case, one would not anticipate a strong correlation between emotion channels, but at the same time, significant performance differences between channels (i.e., an interaction) might be correspondingly more difficult to demonstrate.

More fundamentally, the cognitive status of emotional valence judgments on abstract art remains to be established. Emotional expressions conveyed by faces and voices are largely dissociable from the characteristics of the carrier: a particular face or voice can convey a range of emotions, while conversely a particular emotional expression can be conveyed across the gamut of face and voice identities. It is unlikely that this is the case for artworks: the perceptual configuration of a painting cannot be arbitrarily altered without simultaneously altering the emotional tone of the painting. Emotional judgments on art are likely to entail substantial perceptual decoding that may engage specialised visual and executive mechanisms that may not be comparably engaged in processing other kinds of emotional expression (Ishai et al., 2007, Fairhall and Ishai, 2008, Cupchik et al., 2009). In addition, art as a human artefact is usually intended to engender an aesthetic response: we like paintings to a greater or lesser degree and it is not always clear to what extent this can be separated from the emotional character of the work (Di Dio and Gallese, 2009, Lacey et al., 2011). While these factors dissociate not uncommonly in the case of representational or quasi-representational artworks (we can find *Guernica* beautiful while being simultaneously horrified by its subject matter), the situation is less clear for abstract art. Although not explicit in our task instructions, we therefore acknowledge the possibility that our emotional valence matching paradigm may have recruited components of the aesthetic response to visual art in addition to any mechanism responsible for emotion decoding *per se*.

Impaired processing of emotion from art in our patient cohort was associated with regional grey matter atrophy in right lateral occipital temporal cortex, overlapping the anatomical location of human V5 – MT complex in previous work (Watson et al., 1993, Van Oostende et al., 1997, Annese et al., 2005). This locus is in close proximity to occipito-temporal cortical regions implicated in the processing of kinetic contours (Zeki et al., 2003) and more generally in the assignment of aesthetic value to both representational and nonrepresentational art (Kawabata and Zeki, 2004, Vartanian and Goel, 2004, Cattaneo et al., 2015, Cattaneo et al., 2016). It is of interest that aesthetic appreciations of Richter's work frequently emphasise its ‘kinetic’ quality and analogising the rhythmic and dynamic properties of abstract art as ‘visual music’ has been commonplace since the era of Kandinsky (Spalding, 1980). The present neuroanatomical data suggest that the analysis of dynamic structure may be integral to decoding the emotional content of nonrepresentational art; more broadly, the findings corroborate previous work suggesting that the emotional impact of art may arise from the interaction of posterior cortical perceptual with more anterior evaluative and reward mechanisms (Fairhall and Ishai, 2008, Cupchik et al., 2009, Di Dio and Gallese, 2009, Lacey et al., 2011, Thakral et al., 2012), probably modulated by previous artistic experience (Kim and Blake, 2007). While any interpretation of unthresholded data must be cautious, inspection of parameter maps showing the association between regional grey matter volume and art emotion valence matching performance (Fig. 3) suggests that regional variation of effects encompassed a distributed brain network extending into more anterior temporal and fronto-subcortical areas predominantly in the right hemisphere. This network is vulnerable to FTLD pathologies and its involvement here is in line with previously demonstrated correlates of art emotion processing in the healthy brain (Kawabata and Zeki, 2004, Vartanian and Goel, 2004, Di Dio and Gallese, 2009, Cupchik et al., 2009, Vessel et al., 2012, Aviv, 2014).

From a clinical perspective, our findings illustrate how deficits of emotional awareness in FTLD may transcend the traditional province of neuropsychology to affect complex everyday phenomena such as art with potentially disproportionate significance for patients and families. The findings amplify previous observations of emotion deficits in nfvPPA (Rohrer et al., 2012, Couto et al., 2013), suggesting that such deficits, even if relatively subtle, should still be actively sought in these patients. The data further suggest a need for caution in assuming retained emotional understanding of art in FTLD despite striking and well-documented instances of enhanced artistic output in patients with FTLD (Chatterjee, 2006, Seeley et al., 2008, Miller and Miller, 2013). This apparent paradox may reflect neuropsychological stratification within a notably diverse clinical population; or alternatively, in this respect art may be somewhat analogous to music, from which patients may evidently continue to derive pleasure despite impaired cognitive labelling of emotional states (Omar et al., 2010). These possibilities will only be resolved by detailed examination of art emotion processing in individuals with FTLD who also produce art or at least retain an active interest in it.

This study has limitations that should motivate further work. The valence matching paradigm here raises fundamental questions about the nature of emotional expression in abstract art, its relations to aesthetic experience and the impact of neurodegenerative diseases on these processes. The emerging field of neuroaesthetics has called attention to the distributed brain mechanisms that subserve our sense of beauty and are likely to be vulnerable to neurodegenerative pathologies (Vartanian and Goel, 2004, Ishai et al., 2007, Di Dio and Gallese, 2009, Cattaneo et al., 2015). However, neuroaesthetic studies of patients with dementia remain relatively limited (Chatterjee, 2006, Halpern et al., 2008, Halpern and O'Connor, 2013, Graham et al., 2013, Silveri et al., 2015); future studies should address the components of the aesthetic response in dementia directly, informed by cognitive models that take account of the multi-dimensional nature of aesthetic judgments and the role of potentially confounding perceptual and executive factors (Ishai et al., 2007, Fairhall and Ishai, 2008, Cupchik et al., 2009). The present emotion processing paradigm could be refined in various ways. A requirement for more precise decoding of specific emotions from artworks might help to establish whether a ‘lexicon’ of abstract art-based emotions can be defined, while collection of behavioural rating (and ideally also physiological) data will be required to characterise the subjective emotional experience of art in patients with FTLD. Particularly given the idiosyncratic nature of aesthetic experience, collection of normative data from a larger population of healthy individuals will be essential while the target clinical cohorts should be expanded. It would be of particular interest to compare FTLD with Alzheimer's disease cohorts, in light of emerging evidence that emotional responses to art engage the ‘default mode’ brain network that is targeted by Alzheimer's pathology (Vessel et al., 2012). Larger cohorts would improve power to define functional as well as structural neuroanatomical substrates more robustly. While inferences between healthy and clinical populations always need to be drawn with care, as in other domains of neuropsychology, studying disease populations may reveal neuroanatomical associations that are critical for emotional and aesthetic responses to art. In this sense, information from patients with dementia is complementary to that derived from functional imaging studies of the healthy brain: ideally, patient cohorts would be compared directly with healthy age-matched controls using common functional neuroimaging paradigms, so that network substrates can be fully delineated while at the same time allowing critical network components that support particular functions to be identified (the present data suggest that V5 – MT complex may be one such critical ‘hub’ for decoding abstract art). The boundary between representational and nonrepresentational art is not sharply defined (as exemplified by the cubist creations of Picasso, Braque and others), and this boundary could be explored to compare the cognitive and neural mechanisms that decode emotions from abstract and animate stimuli. It would also be of interest to compare emotion processing in visual art and music directly. Studies equipped to probe the interface between emotion modalities should allow us to resolve the extent to which mechanisms of emotion decoding are shared between emotion channels or may be relatively specific to particular modalities. The present findings provide a preliminary rationale for a more detailed and extensive examination of emotion decoding from art and other abstract symbolic patterns in neurodegenerative disease.

## Figures and Tables

**Table 1:** Demographic, clinical and general neuropsychological characteristics of participant groups.

**Characteristic**	**bvFTD**	**svPPA**	**nfvPPA**	**Healthy controls**
**Demographic**				
No. (M:F)	11 (9:2)	7 (5:2)	6 (3:3)	39 (18:21)*
Handedness (R:L)	10:1	7:0	5:1	36:3
Age (years)	68 (8.6)	64 (7.8)	68 (8.7)	68 (7.6)
Education (years)	17 (3)	13 (3)^a,b^	**17 (0)**	16.1 (2.1)
Art experience (0–5 scale)^†^	1.7 (1.3)	1.6 (1.3)	1.8 (0.8)	1.9 (1.6)
Symptom duration (years)	8.5 (5.3)	6.1 (2.3)	3.2 (1.5)^b,c^	N/A
MMSE (/30)	25.1 (2.8)	26.0 (2.2)	25.5 (5.7)	N/A
**General neuropsychological functions**				
**General intellect**				
WASI Vocab (/80)	**51 (13)**	**41 (14)**	**43 (15)**	71 (4)
WASI Blocks (/71)	**26 (12)**	40 (20)	**20 (19)**	42(15)
WASI Similarities (/48)	**23 (10)**	**21 (9)**	**28 (6)**	39 (5)
WASI Matrices (/32)	**18 (6)^c^**	25 (6)	21 (6)	25 (5)
NART Total (/50)^d^	33 (11)	**26 (11)**	33 (13)	42 (5)
**Executive skills**				
D-KEFS Stroop colour (seconds)^e^	**44 (12)**	43 (12)^b,c^	76 (24)^,^	28 (8)
D-KEFS Stroop word (seconds)^e^	**29 (8)**	24 (6)	47 (19)	22 (4)
D-KEFS Stroop inhibition (seconds)^e^	**98 (38)^a^**	78 (29)^a^	**156 (23)**	59 (21)
Trails A (seconds)	60 (27)	38 (19)	69 (39)	35 (11)
Trails B (seconds)	**195 (84)**	98 (43)^a,b^	**189 (67)**	87 (41)
WAIS-R Digit-Symbol (/90)	**31 (7)**	41 (11)	**27 (11)^c^**	54 (11)
Letter Fluency^e^	**9 (4)**	**10 (5)**	**4 (3)**	17 (5)
Category Fluency^e^	**10 (5)**	**5 (3)**	**9 (4)**	22 (7)
**Visual perception**				
VOSP Object Decision (/20)^f^	17 (2)	17 (3)	17 (3)	18 (2)
Hue discrimination (/48)^g^	47 (45–48)	48 (48–48)	**45** (43–48)	47.9 (0.2)
**Emotion recognition**				
Adapted Ekman faces (/40)^h^	**31** (27–34)	36 (34–39)	**32** (27–37)	37.6 (1.4)
**Episodic memory**				
RMT Faces (/50)^i^	**30 (12)**	37 (7)	37 (7)	44 (4)
RMT Words (/50)^j^	**34 (15)**	**37 (4)^a^**	47 (3)	48 (2)
Camden Paired Associate Learning (/24)^f^	**8 (7)^a^**	**4 (4)^a^**	19 (4)	20 (3)
**Language skills**				
BPVS (/150)	137 (12)	112 (34)	145 (5)	147 (3)
GNT (/30)^d^	**9 (9)^a^**	**0.1 (0.4)^a,b^**	**9 (2)**	25 (3)
Polysyllabic word repetition (/45)	N/A	43 (3.3)	27 (21)	N/A^k^
**Other skills**				
WMS-R Digit Span Forward (/12)^d^	8.6 (2.6)	9.3 (2.0)	8.0 (1.4)	9.0 (1.9)
WMS-R Digit Span Reverse (/12)^d^	6.4 (2.3)	8.7 (3.1)	5.5 (2.1)	7.7 (2.1)
GDA Addition (/12)^d^	6 (3)	6 (3.5)	3 (1)	7 (3)
GDA Subtraction (/12)^d^	5 (3)	5 (5)	4 (4)	8 (3)

Mean (standard deviation) values are presented unless otherwise indicated; maximum scores on neuropsychological tests are given in parentheses. Key: statistically significant differences from healthy control values (*p*<0.05) indicated in bold; *a subset of 25 individuals completed the background neuropsychological assessment; †see text and Table S2 on-line for details; ^a^, significantly lower than nfvPPA group (*p*<0.05); ^b^, significantly lower than bvFTD group (*p*<0.05); ^c^, significantly lower than svPPA group (*p*<0.05); ^d^, two patients with nfvPPA unable to attempt this test; ^e^, three patients with nfvPPA unable to attempt this test; ^f^, one patient with nfvPPA unable to attempt this test; ^g^, ranges shown for patient performance (five bvFTD, three svPPA, three nfvPPA), referenced to historical group of 54 healthy older controls (Shakespeare et al., 2013); ^h^, ranges shown for patient performance (five bvFTD, three svPPA, three nfvPPA), referenced to historical group of 21 healthy older controls (Omar et al., 2011a); ^i^, two patients with bvFTD unable to complete this test; ^j^, three patients with bvFTD unable to complete this test; ^k^, healthy native speakers assumed to be at ceiling for this test; BPVS, British Picture Vocabulary Scale; BPVS, British Picture Vocabulary Scale; bvFTD, behavioural variant frontotemporal dementia; D-KEFS, Delis-Kaplan Executive Functioning System; GDA, Graded Difficulty Arithmetic test; GNT, Graded Naming Test; MMSE, Mini-Mental State Examination score; N/A, not available; NART, National Adult Reading Test; nfvPPA, nonfluent – agrammatic variant of primary progressive aphasia; RMT, Recognition Memory Test; svPPA, semantic variant of primary progressive aphasia; VOSP, Visual Object and Space Perception Battery; WAIS-R, Wechsler Adult Intelligent Scale Revised; WASI, Wechsler Abbreviated Scale of Intelligence; WMS-R, Wechsler Memory Scale Revised.

**Table 2:** Summary of participant group performance on experimental tests.

**Test**	**bvFTD**		**svPPA**		**nfvPPA**		**Healthy controls**	
***Emotion matching task^⁎^***								
Art emotion (/20)	11.6 (3.2)	*(58%)*	12.6 (2.5)^⁎^^⁎^	*(63%)*	13.0 (0.9)^⁎^	*(65%)*	15.7 (2.3)	*(79%)*
Facial emotion (/24)	14.3 (3.1)	*(60%)*	18.6 (3.0)	*(78%)*	15.5 (3.9)^⁎^	*(65%)*	20.2(2.2)	*(84%)*
***Perceptual control task***								
Art perception (/20)	18.2 (1.5)	*(91%)*	18.6 (1.0)	*(93%)*	17.8 (1.0)^**^	*(89%)*	18.9 (0.9)	*(95%)*

Mean (standard deviation) raw values are presented, followed by mean percent correct in italics; maximum scores on experimental tests are given in parentheses. Key: *all patient groups significantly impaired (*p*<0.05) relative to healthy controls but no significant interaction with emotion modality (see text for details); **significantly impaired (*p*<0.05) relative to healthy control group; bvFTD, behavioural variant frontotemporal dementia; nfvPPA, nonfluent – agrammatic variant of primary progressive aphasia; svPPA, semantic variant of primary progressive aphasia.

## Footnotes

Appendix A Supplementary data associated with this article can be found in the online version at doi:10.1016/j.neuropsychologia.2015.12.031.
