# Supplementary material for: Processing emotion from abstract art in frontotemporal lobar degeneration
Source: Neuropsychologia. 2016 Jan 29;81:245–54. doi: 10.1016/j.neuropsychologia.2015.12.031 (PMC4749539; doi:10.1016/j.neuropsychologia.2015.12.031)
Supplement: Supplementary file 1 — Supplementary material [file mmc1.docx]

**SUPPLEMENTARY MATERIAL**

**Processing emotion from abstract art in frontotemporal dementia, by MH Cohen et al**

**Details of stimuli used in art emotion valence and perceptual matching tests**

High-quality digital reproductions of Richter abstract paintings were obtained from publically accessible on-line libraries and converted to JPEG images each with dimensions either 80 x 80 mm (probe stimuli) or 60 x 60 mm (targets and foil stimuli) and resolution 300 dpi. These images were arranged into triads for the emotion matching and perceptual matching tests as described in the text (see also Table S1 below) and presented from the monitor of a Dell Latitude computer (screen resolution 1366 x 768 pixels) running Powerpoint®v14 with 24-RGB colour scale; images were presented at a comfortable viewing distance under natural lighting on a plain white background with the configuration shown in Figure 1. On each trial, the probe stimulus was always presented above and the target and foil stimuli (labelled A and B) below with their respective positions randomly interchanged between trials. Successive trials were triggered by the examiner following an untimed participant response.

**Table S1. Stimuli used in the art emotion test** [all works by Gerhard Richter]

| **Trial** | **Probe** | **Target** | **Foil** | **Probe valence** |
| --- | --- | --- | --- | --- |
| 1 | 1997, 846-2 | **B** 1987, 648-4 | **A** 1999, 862-7 | P |
| 2 | A, B Sanctuary (1988), 655 | **A** 2006, 989-12 | **B** 1999, 858-3 | N |
| 3 | 2009, 907-12 | **B** 2008, 906-4 | **A** 1997, 845-3 | N |
| 4 | A, B Courbet (1986), 616 | **A** 1995, 829-2 | **B** See, 1997, 848-1 | P |
| 5 | 1990, 725-5 | **B** 2009, 908-5 | **A** 2009, 910-1 | N |
| 6 | St John, 1988 | **B** 1988, 675-9 | **A** 1990, 724-4 | N |
| 7 | 1999, 858-3 | **A** 1999, 858-4 | **B** 2006, cage painting | P |
| 8 | 1995, 825-5 | **A** A, B Sanctuary (1988), 655 | **B** 2001,873-7 | N |
| 9 | 2009, 910-1 | **B** 1999, 858-3 | **A** 2004, 890-6 | P |
| 10 | 1985, 575-1 | **A** 1997, 845-3 | **B** 1992, 778-4 | P |
| 11 | 1987, 648-4 | **B** 1999, 858-1 | **A** 2008, 903-6 | P |
| 12 | 1999, 858-1 | **A** 1995, 829-2 | **B** 1990, 725-5 | P |
| 13 | 2006, 898-12 | **A** 1986, 590 | **B** 1986, A, B Courbet, 616 | N |
| 14 | 1998, 850-1 | **A** 2009, 910-4 | **B** 1999, 862-7 | P |
| 15 | 1992, 769-1 | **B** 1985, 575-1 | **A** 1988, 675-9 | P |
| 16 | 1997, 843-9 | **A** 1995, 825-5 | **B** 1999, 858-14 | N |
| 17 | See, 1997, 848-1 | **B** 2006, 898-12 | **A** 1999, 858-4 | N |
| 18 | 1990, 726 | **B** St-John, 1988 | **A** 2009, 910-4 | N |
| 19 | 1988, 675-9 | **A** 1999, 862-4 | **B** 1990, 724-4 | N |
| 20 | 1997, 845-3 | **B** 1998, 850-1 | **A** 1986, 590 | P |

Works by Gerhard Richter are identified by Catalogue Raisonné number (descriptive titles given where supplied). Images can be found at the artist’s website: <https://www.gerhard-richter.com/en/>. Trials were presented in randomised order during the test and screen positions (**A**, **B**) of target and foil images were randomised between trials, N, negative; P, positive

**Table S2. Art experience questionnaire**

1)      Have you ever had any artistic training? (e.g. lessons, art classes, courses, etc ; art classes at lower-level in junior school excluded ) YES   NO

If yes (*scores 1 point)*:

1a) What kind of training and for how long?

2) Have you ever produced your own artwork? (Painting, drawing, sculpting, etc.)   YES   NO

 If yes (*scores 1 point)*:

2a) Which medium(s) do you use?

2b) Would you describe your artwork as abstract, representational or semi-representational?

If answer YES to Question 2:

3)      Do you still produce art regularly? YES   NO

If yes *(scores 1 point)*:

3a) What kind of artwork? (medium, subject)

3b) Approximately how often do you produce artwork?

3c) Is this in a taught environment or at home?

4)      Do you view professional artwork regularly? (e.g., visiting exhibitions) YES   NO

If yes *(scores 1or 2 points)*:

4a) How often do you do this?

*1 - 6 times per year – 1 point; >6 times per year – 2 points.*

5) What kind of artwork do you most enjoy? (Style, medium, movements, favourite artists)

 6)      Can you identify the artist whose work was used in this test?

*Art background was scored on a scale between 0 and 5 (0 indicating no previous interest or art experience, 5 indicating very high level of interest, experience, or professional artist). A modified version of the questionnaire was completed by patients’ caregivers.*


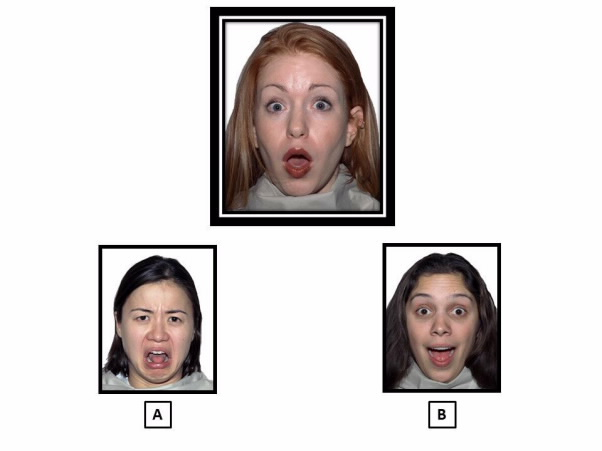


**Figure S1**. An example of a stimulus triad from the facial emotion matching test (facial expression stimuli adapted from the NimStim set: Tottenham et al., 2009). The probe stimulus is shown above; the foil and target stimuli are below. In this example, the probe stimulus represents surprise; the target (matching) stimulus here is B.


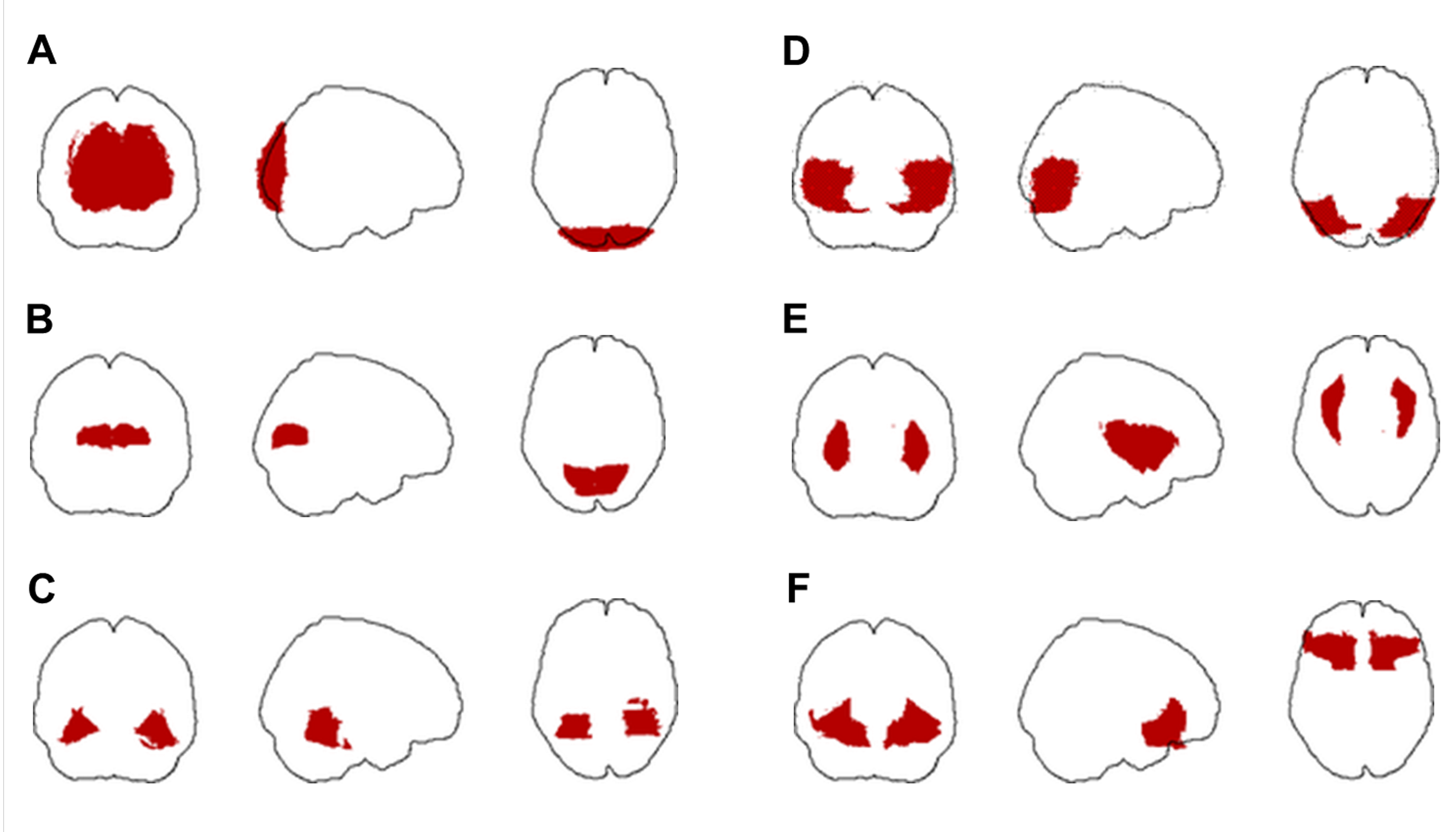


**Figure S2.** Anatomical regions of interest used for small volume correction in the present study covered primary visual and ventral visual association cortices (**A,B,C**), dorso-lateral and occipito-temporal association cortices (**D**) insula (**E**) and orbitofrontal cortex (**F**) in both hemispheres. Regions were derived from the Harvard-Oxford brain maps, shown here projected on the MNI single subject glass brain (adapted from: http://qnl.bu.edu/obart/region/HO/). Comparing posterior visual regions with a probabilistic histological brain atlas (http://qnl.bu.edu/obart/compare/HO/CYTO/) suggests maximal overlap with individual visual cortical areas as follows: V1, **A,B**; V2, **A,B**; V3, **A**; V4, **C**; V5, **D**. Regions in each cerebral hemisphere were assessed separately


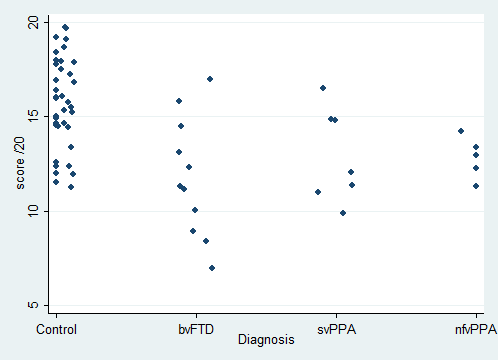


**Figure S3.** Plots of raw individual scores (% correct) on the art emotion valence matching test for participants in each of the experimental groups. A score of 10 (50% correct) corresponds to chance level performance. Key: bvFTD, behavioural variant frontotemporal dementia; nfvPPA, nonfluent – agrammatic variant of primary progressive aphasia; svPPA, semantic variant of primary progressive aphasia
